# Evaluation of Polyphenolic Compounds and Pharmacological Activities in Hairy Root Cultures of *Ligularia fischeri* Turcz. f. *spiciformis* (Nakai)

**DOI:** 10.3390/molecules24081586

**Published:** 2019-04-22

**Authors:** Mohammad Azam Ansari, Ill-Min Chung, Govindasamy Rajakumar, Mohammad A. Alzohairy, Ahmad Almatroudi, Venkatesan Gopiesh Khanna, Muthu Thiruvengadam

**Affiliations:** 1 Department of Epidemic Disease Research, Institutes for Research and Medical Consultations (IRMC), Imam Abdulrahman Bin Faisal University, Dammam 31441, Saudi Arabia; maansari@iau.edu.sa; 2 Department of Applied Bioscience, College of Life and Environmental Sciences, Konkuk University, Seoul 05029, Korea; imcim@konkuk.ac.kr (I.-M.C.); microlabsraj@gmail.com (G.R.); 3 Department of Medical Laboratories, College of Applied Medical Sciences, Qassim University, Qassim 51431, Saudi Arabia; dr.alzohairy@gmail.com (M.A.A.); aamtrody@qu.edu.sa (A.A.); 4 Department of Biotechnology, School of Life Sciences, Vels Institute of Science, Technology and Advanced Studies (VISTAS), Vels University, Pallavaram, Chennai 600117, Tamil Nadu, India; gopieshkhanna.sl@velsuniv.ac.in

**Keywords:** *Ligularia fischeri*, *Agrobacterium rhizogenes*, hairy root cultures, polyphenolic compounds, pharmaceutical activities

## Abstract

A considerable amount of bioactive compounds have been used for the biopharmaceutical engineering to help human health and nutrition. Hairy root culture (HRC) or transgenic root is a favourable alternative technique for phytochemical production. *Ligularia fischeri* is a significant source of pharmaceutically important active compounds with an enormous range of health care applications. HRC of *L. fischeri* was developed using *Agrobacterium rhizogenes* for the production of polyphenolic compounds with antioxidant, antimicrobial, antidiabetic, anticancer and anti-inflammatory pharmaceutical activities. Hairy roots (HRs) were selected by morphological assessment, genetic and molecular analyses. The maximum accumulation of fresh mass (94.15 g/L) and dry mass (9.45 g/L) was recorded in MS liquid medium supplemented with 30 g/L sucrose at 28 days. Furthermore, HRs successfully produced numerous polyphenolic compounds, including six hydroxycinnamic acids, seven flavonols, seven hydroxybenzoic acids, vanillin, resveratrol, pyrogallol, homogentisic, and veratric acids, which were identified by UHPLC analysis. HRs produced higher total phenolic (185.65 mg/g), and flavonoid (5.25 mg/g) contents than non-transformed roots (125.55 mg/g and 3.75 mg/g). As a result of these metabolic changes, pharmaceutical activities were found higher in HRs than non-transformed roots (NTRs). The present study indicates that HRC has the potential to increase the content of beneficial polyphenolic compounds with higher potential pharmaceutical activities. To the best of our knowledge, the present study is the first report on enhancing the production of polyphenolic compounds with pharmaceutical activities from the HRCs of *L. fischeri*.

## 1. Introduction

The *Ligularia*, which belong to the Senecioneae tribe (Family: *Asteraceae*) comprise about 140 species of perennial herbs. *Ligularia fischeri* is a leafy vegetable that is widely distributed in wet, shady areas of Korea, Japan, China, Europe, and Eastern Siberia. It is also called *Gomchi* in Korean, and the leaves are consumed as a fresh vegetable. *Ligularia fischeri* has been used in traditional Chinese medicine for hundreds of years owing to its reported curative power for bacterial infections, rheumatism, bronchitis, coughing, tumour, asthma, hemoptysis, pulmonary tuberculosis, and hepatitis [1]. In Korean folk medicine, it has been used for the treatment of prolonged alcohol use, hepatitis, jaundice, cancer, inflammation, cough, scarlet-fever, emptysis, hemoptysis, diuresis, and rheumatoid arthritis. Previous studies have shown that *Ligularia* species contain a variety of phytochemicals with interesting biological activities. *L. fischeri* roots contain pharmaceutically important bioactive compounds such as protocatechualdehyde, β-hydroxychromone, caffeic acid, ferulic acid, 1,5-dicaffeyolquinic acid, angelic acid, β-sitosterol, daucosterol, petasin, isopetasin, and pentatriacontane used for promoting blood circulation, in the treatment of coughs and for relieving pain [2]. Earlier reports showed that the leaves contained bioactive compounds such as sesquiterpenoids, phenolic compounds, terpenoids, spiciformisins, monocyclosqualene, caffeoylquinic acid and norsesquiterpene derivatives [1,3,4]. According to the prior literature, *L. fischeri* is a significant plant due to its multiple pharmaceutical activities. 

Polyphenols have attracted much interest because of their powerful pharmacological potential and many uses in medicine [5]. Polyphenols are bioactive compounds, found mostly in various plants, vegetables, fruits, whole grains, nuts, beverages, olive oil and are known as the most common substances which possess multiple ranges of biomedical uses [6]. Polyphenols are divided into four different groups based on the presence of some phenol rings and structural components such as phenolic acids, flavonoids, stilbenes, and lignans [7]. The regular intake of polyphenol-rich foods may help decrease the risk for cardiovascular diseases, colon cancer, liver disorders, obesity and diabetes [8]. It has been revealed that phenolic compounds have great pharmaceutical potential such as anti-thrombosis, anticancer, antivirus, antioxidant, antihypertension, anti-inflammatory activities [9,10,11]. Phenolic and flavonoids have attracted more attention owing to their strong pharmaceutical potential and various medical applications such as potent antioxidant, free radical scavengers, and metal chelators, anticholinesterase, antiaging, neuroprotective, anti-inflammatory, antidepressant and anti-amyloidogenic properties [5,12]. A meta-analysis reported that flavonoids and lignin are associated with decreased risk of all-cause mortality, cancers, cardiovascular and neurodegenerative diseases [6]. Dietary consumption of flavonoids, namely flavonols, anthocyanidins, proanthocyanidins, flavones, flavanones and flavan-3-ols, significantly decreases the risk of cardiovascular diseases [13]. Nutritional intakes of flavonoids are connected with a decreased risk of breast, prostate, colorectal, ovarian, esophageal, gastric, and lung cancers [6]. Recent, investigations reported that some plant-derived polyphenol compounds have neuroprotective potential for the treatment of neurodegenerative diseases. Flavonoids are stated to have potential for the treatment of neurodegenerative diseases such as Parkinson’s and Alzheimer’s diseases [12]. The demand for polyphenolic compounds extracted from *L. fischeri* roots has led to uncontrolled uprooting, harmfully disturbing the plant in its natural habitat. The amounts of phytochemicals in harvested field-grown plants are unbalanced as the plants are exposed to different environmental and biotic factors. Therefore, alternative methods must be identified to produce these phytochemicals.

*Agrobacterium rhizogenes* is an innate Gram-negative soil bacterium that incites hairy root (HR) growth in many plants. The root loci (*rol*) genes contained in this bacterium’s root induction (Ri) plasmid are transferred and combined in the host plant genome, causing HR. Further, it results in negative geotropic, extensive branched and well-grown roots. HRs are distinctive in terms of their genetic and biosynthetic constancy and have been intensively used to induce a continuous and large scale production of specific phytochemicals [14]. Recently, hairy root cultures (HRCs) have become a useful biological system to study the biosynthesis of bioactive compounds such as gymnemic acid, anthraquinones and phenolic compounds [10,14,15]. *RolC* transgenic *Artemisia carvifolia* improved the content of artemisinin (sesquiterpene), and it has used for anti-malaria and anti-cancer activities [16]. Furthermore, *rolC* increases antioxidant, anticancer, antimicrobial, anti-analgesic and anti-inflammatory properties in lettuce [17] and *Momordica dioica* [18]. This is the first report on the transformation of *L. fischeri* with *A. rhizogenes* for the production of bioactive compounds and their pharmacological potential. This investigation aimed to grow a fast-growing HRC system to facilitate the production of pharmaceutically significant polyphenolic compounds. We have also assessed the total phenolics, flavonoids and phenolic compounds (seven flavonols, six hydroxycinnamic acids, seven hydroxybenzoic acids, homogentisic acid, vanillin, and resveratrol) and their pharmacological potential (antioxidant, antimicrobial, antidiabetic, anticancer and anti-inflammatory activities) in HRCs of *L. fischeri*.

## 2. Results and Discussion

### 2.1. Formation of Transgenic Hairy Root Cultures (HRCs)

**Figure 1:**
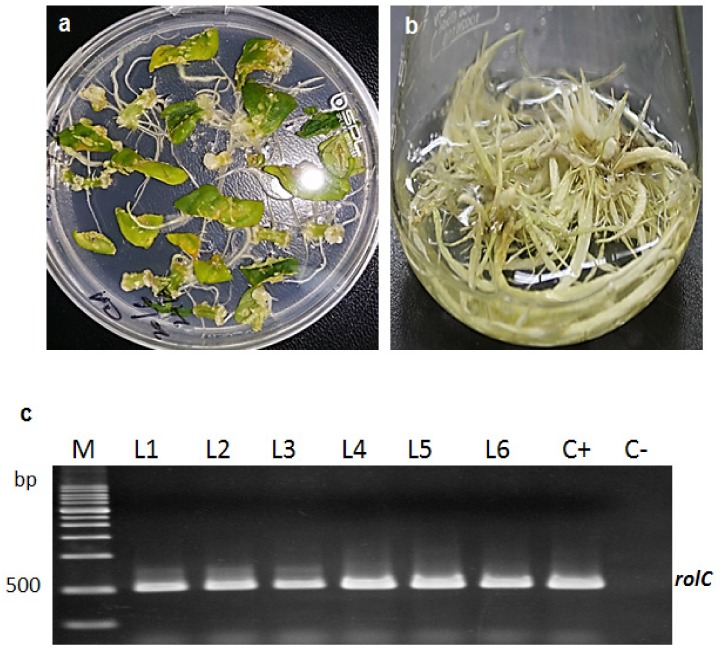
*Agrobacterium rhizogenes*-mediated hairy root cultures in *Ligularia fischeri*. (**a**) Hairy roots induction, (**b**) Hairy root cultures in hormone-free liquid MS medium, **c.** PCR analysis of the *rolC* gene in the transgenic root lines. DNA ladder marker lane M, pRiKCTC2703 DNA C(+), transgenic root lines (HRs) induced by *A. rhizogenes* L1–L6, roots from a non-transgenic plant (NTRs) C(−).

The leaf, petiole, and roots were inoculated with *A. rhizogenes* (KCTC 2703) for hairy root (HR) induction. Leaf explants produced more HRs (81%) than did petiole (12%) explants, whereas root explants did not respond within the 15 day culture. This experiment proved that leaf explants are suitable for HR induction in *L. fischeri*. Moreover, explant selection is an essential factor in the successful development of HRs [19]. In agreement with our results, a higher frequency of transformation from leaves was reported in *Semecarpus anacardium* [19] and *Polygonum multiflorum* [20]. Initial HRs arose from the wounded parts of the leaves and petioles within 12–15 day after co-cultivation. After 21–23 day, HRs of *L. fischeri* began to grow more rapidly (Figure 1a). Observations showed that the HRs had a high rate of extensive lateral branching, the absence of geotropism and produced a greater abundance of HRs than NTRs. Similar characteristics of HRs resulting from *A. rhizogenes* were reported in several plants [21]. No roots or calli was observed from the explants of non-infected non-transgenic (NT) explants. HRs appeared at the wounding site of explants, and so, it may have been easier for *A. rhizogenes* to infect leaf explants, in which the wounded place is a vast area and, therefore, present the T-DNA inducing HR to the plant genome. The results achieved exhibit the same results [21]. The phloem cells, located inside the midrib region are a target of *A. rhizogenes* [22]. HRs were excised from the explants and inoculated in the MS liquid medium with cefotaxime for suspension culture. About 28 day was required for the full development of HRs in MS liquid medium (Figure 1b). Six rapidly growing putative transgenic HR lines and NTRs have been randomly selected. DNA has been obtained from HR and NTRs. PCR amplification using a *rolC* primer with a fragment of 500 bp confirmed the HRs. The fragment for *rolC* was noticed in the amplified DNA from all the six transgenic HR lines (line 1–6; Figure 1c) while no fragment was observed for control NTR (C−; Figure 1c). *A. rhizogenes* KCTC 2703 was used as a positive control (C+; Figure 1c). The *rol* genes play a vital role in the development of HRs and also in the production of bioactive compounds owing to the integration of the T-DNA region of the root-inducing (Ri) plasmid into the host genome [23]. *rolC* is the most attractive gene for biotechnological studies since it is proficient of stimulating both the growth of transformed cells and the biosynthesis of phytochemicals [10]. The *rolC* may stimulate the accumulation of tropane alkaloids in HRs of *Atropa belladonna* [24], phenolic compounds in *Polygonum multiflorum* [10,20], and caffeoylquinic acid accumulation in transformed artichoke cells [25].

### 2.2. Factors Influencing the Growth Kinetics in Biomass Accumulation

**Figure 2:**
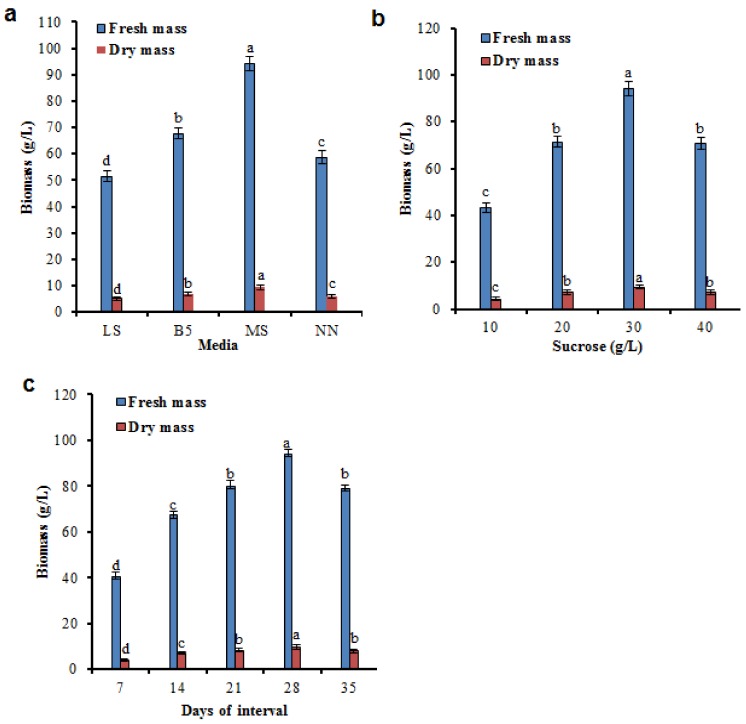
Factors influencing the biomass accumulation in hairy root cultures (HRCs) of *Ligularia fischeri*. (**a**) HRCs as affected by different media, (**b**) HRCs as affected by different concentrations of sucrose in the MS medium. (**c**) Time profile of HRCs in MS medium supplemented with sucrose (30 g/L). Data represent as means ± SD of three replicates. If followed by different letters, results are significantly different at *p* ≤ 0.05.

The accumulation of biomass differed significantly among the various media in HRCs (Figure 2a). Sucrose is the vital source of carbohydrate which is used as the chief source of energy for biomass accumulation (Figure 2b). In line with our results, MS medium with sucrose (30 g/L) preferred biomass and the accumulation of phenolic compounds in HRCs [26]. The time passage study inferred that HR growth included four different stages, including a lag phase (0–7 day), an exponential phase (7–28 day), a stationary phase (28–35 day), and a death phase (after 36 day). Maximum biomass production (94.15 g/L FM and 9.45 g/L DM) was attained in MS liquid medium appeared near the end of exponential phase at exactly 28 d of culture (Figure 2c). 

**Figure 3:**
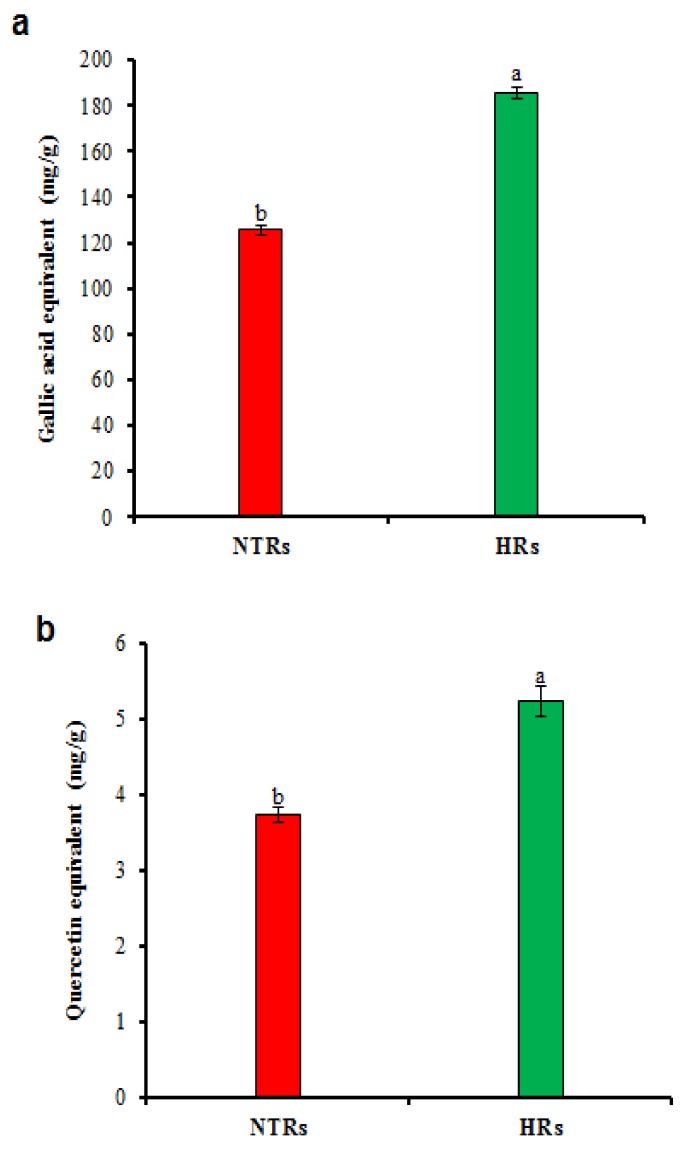
Total phenolic and flavonoid contents (TPC and TFC) in hairy root cultures of *Ligularia fischeri*. (**a**) TPC, (**b**) TFC. Data represent as means ± SD of three replicates followed by different letters, are significantly different at *p* ≤ 0.05.

Corresponding to our results, 28 day of culture HRs produced a high amount of biomass and phytochemical accumulations in *Sphagneticola calendulacea* [27]. The present study, HRCs established in MS medium with 30 g/L sucrose showed the maximum production of biomass, and phenolic compounds were observed at 28 day. HRC lines showed the maximum concentration of phenolic compounds, total phenolic and flavonoid contents than non-transformed roots (NTRs) of *L. fischeri* (Table 1; Figure 3a,b). The results suggested that HRCs are auspicious for maximum production of biomass and phenolic compounds in liquid cultures.

### 2.3. Polyphenolic Contents 

UHPLC simultaneously detected twenty five polyphenolic compounds, including seven flavonols (myricetin, naringenin, quercetin, kaempferol, rutin, biochanin A, and formononetin), six hydroxycinnamic acids (ferulic, caffeic, chlorogenic, *p*-coumaric, *m*-coumaric and *o*-coumaric acids), seven hydroxybenzoic acids (gallic, gentisic, protocatechuic, *β*-resorcylic, vanillic, syringic, and salicylic acids) and five other phenolic compounds (vanillin, resveratrol, pyrogallol, veratric and homogentisic acids). HRs contained significantly higher flavonols (3198.35 μg/g), hydroxycinnamic acid (558.70 μg/g), hydroxybenzoic acid (776.94 μg/g) and other phenolic compounds (271.20 μg/g) compared to non-elicited HRs at 2596.35, 396.80, 552.90 and 197.80 μg/g, respectively (Table 1). Correspondingly, flavonoids and phenolic acids content were higher in HRs than NTRs of *Hypericum perforatum* [28] and *Momordica dioica* [18]. The level of rutin, quercetin, kaempferol, caffeic, protocatechuic, gallic, ferulic and chlorogenic acids were higher in HRs than NTRs [29]. Like in our results, caffeic, 4-hydroxybenzoic, vanillic, chlorogenic, *p*-coumaric, sinapic, ferulic, trans-*o*-hydroxy-cinnamic and trans-3-hydroxy-4-methoxycinnamic acid levels were significantly raised in HRs compared to NTRs of *Linum usitatissimum* [30]. Quercetagetin, quercetin, luteolin, patuletin, chlorogenic and caffeoylquinic acid derivatives were more elevated in HRs than NTRs of *Rhaponticum carthamoides* [31]. Chlorogenic acid derivatives were raised in HRs compared to NTRs of *Stevia rebaudiana* [32]. The content of flavonoids, hydroxycinnamic acids in the HRs was several times higher than their level in the intact plant roots of *Nitraria schoberi* [33]. In contrast, HRs and NTRs had a similar amount of phenolic compounds in *Platycodon grandiflorum* [34]. The contents of quercetin caffeic and chlorogenic acids were higher in HRs than NTRs of *Fagopyrum tataricum* [35]. Contrastingly, accumulation of *o*-coumaric acid was lower in the HRs than in the NTRs. Similarly, the content of kaempferol was lower in the HRs than NTRs of *Sphagneticola calendulacea* [27]. Caffeoylquinic acids and their derivatives were 2–3-times higher in the wild grown NTRs than the HRs [31]. Reduction in the amount of *o*-coumaric acid and an increase in the other compounds may be caused by feedback inhibition of some metabolic pathways in the HRs. An analogous phenomenon has been demonstrated [36], where thebaine content was decreased while codeine and morphine contents were raised in the HRs.

The phenolic and flavonoid contents in HRs and NTRs were assessed. We found considerable differences in TPC and TFC between HRs and NTRs. TPC showed higher levels (185.65 mg/g GAE) in HRs than NTRs (125.55 mg/g GAE) (Figure 3a). TFC showed a significantly higher content (5.25 mg/g QE) compared to NTRs (3.75 mg/g QE) (Figure 3b). Higher amounts of TPC and TFC in HRs could be due to the effect of *rolC* that up-regulates the genes involved in phytochemical production [37]. The TPC in HRCs showed 3.5-fold higher levels than in NTRs of *Linum usitatissimum* [30]. HRs accumulated higher phenolics, flavonoids and quercetin contents compared to NTRs of radish [38]. Polyphenolic biosynthetic genes (*CHS, FLS*, *CHI*, and *PAL*) showed higher levels in the HRs than in NTRs of *Brassica rapa* [37] and *Lactuca serriola* [39]. The accumulations of a higher amount of TPC and TFC in the HRs could be due to the effect of *rolC* that up-regulates the genes involved in phytochemical production as reported in many other plant species [10,39,40]. Environmental factors, including wound and bacterial infection, increase the biosynthesis of phenolic compounds [41]. Therefore, the *rolC* gene and environmental factors have noteworthy effects on the content of phenolic compounds.

### 2.4. Pharmacological Activities

**Figure 4:**
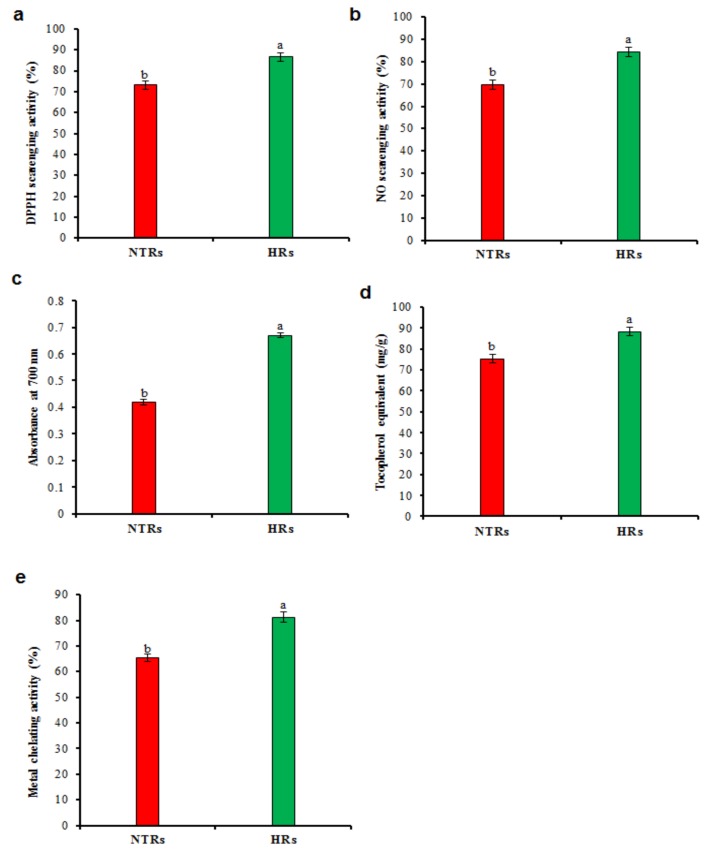
Antioxidant activities in hairy root cultures of *Ligularia fischeri*. (**a**) Percentage inhibition of DPPH radicals, (**b)** Nitric oxide (NO) scavenging activity, (**c**) Total Fe^3+^–Fe^2+^ reductive potential reference antioxidants (butylated hydroxytoluene), (**d**) Total antioxidant activities by phosphomolybdenum method [expressed as equivalents of α-tocopherol (μg/g of extract)], (**e**) Metal chelating activity. Data represent means ± SD of three replicates. If followed by different letters, values are significantly different at *p* ≤ 0.05.

Autoxidation of unsaturated lipids in foods and oxidative cell damage is caused by free radicals thereby causing various diseases in human beings [42]. A strong positive correlation was found among all improved bioactive compounds and antioxidant activities in HRs. The comparative assessment of antioxidant activity was carried out for HR and NTR extracts. Results exhibited substantial improvement in antioxidant activity for HR than that of NTRs. HR extracts displayed the highest DPPH radical scavenging activity which is 86.55% increased than NTRs 73.25% (Figure 4a). Nitric oxide (NO) is a significant bio-regulatory molecule in the nervous, immune and cardiovascular systems [43]. HR extracts showed NO scavenging activity of 84.25% than NTR extracts 69.55% (Figure 4b). Similar to our results, free radical scavenging assays showed higher antioxidant activity in HRs as compared to NTRs in *Linum usitatissimum* [30]. 

The obtained results suggest that reducing capacity HRs have more significant potential than NTRs (Figure 4c). Consistently, reducing power was higher in the HRs as compared to the NTRs in *Lactuca serriola* [39]. HR extracts were stronger reducers of metal ions than the extract of NTRs. Antioxidant activity of the HR extract was 88.25 mg/g, and NTR extract was 75.15 mg/g (Figure 4d). Figure 4e displays the metal scavenging activity of HRs (81.25%) was higher than NTR extracts (65.5%). This increasing antioxidant potential could be recognised to the high phytochemicals formed in the HRs [17]. Similarly, the HRs were showed of greater antioxidant potential in *Lactuca sativa* [17] *L. serriola* [39] and *Linum usitatissimum* [30]. 

**Figure 5:**
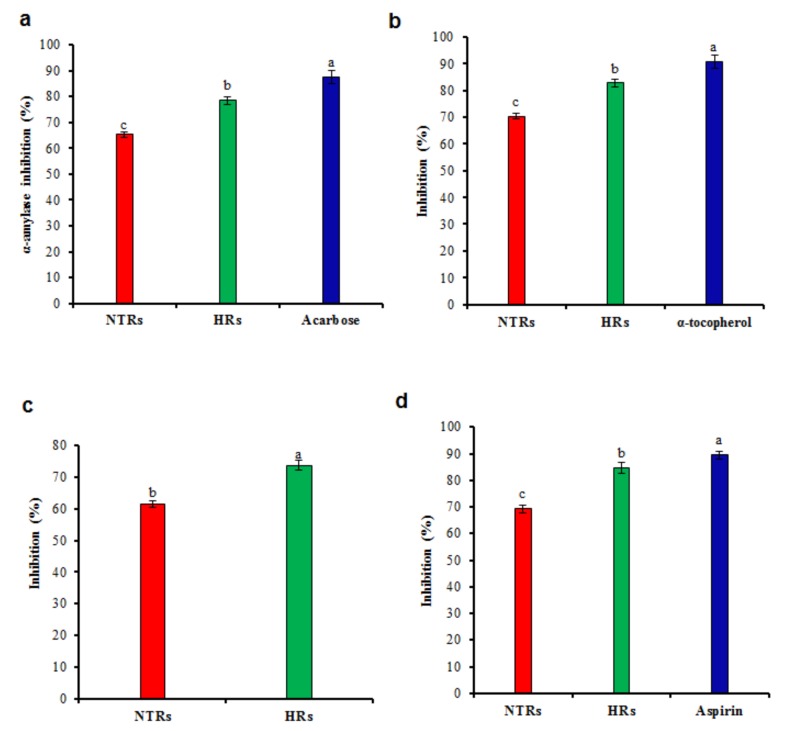
Antidiabetic and anti-inflammatory activities in hairy root cultures of *Ligularia fischeri*. (**a**) In vitro α-amylase activity, (**b**) Non-enzymatic glycosylation of hemoglobin activity, (**c**) Lipoxygenase inhibition activity, (**d**) Albumin denaturation inhibition assay. Data represent as means ± SD of three replicates. If followed by different letters, values are significantly different at *p* ≤ 0.05.

Free radical scavengers are used to manage the oxidative damage and to control enzymes like α-amylase and α-glucosidase which are responsible for causing diabetes [44]. The enzyme α-amylase is used for the intestinal digestion process that hydrolyses polysaccharides to simple monosaccharides, therefore playing a dominant role in carbohydrate digestion [43]. In this study, the activity of the α-amylase enzyme was expressively inhibited in HR extracts (Figure 5a). The results displayed that α-amylase was dramatically suppressed in a concentration-dependent manner after incubation with various concentrations of extracts. The HR extracts (100 μg/mL) exhibits 78.55% and NTR 65.25% of α-amylase enzyme inhibition (Figure 5a). In the meantime, acarbose exhibited 87.50% of inhibition (Figure 5a). Inhibition of α-amylase can lead to a decrease in postprandial hyperglycemia. Anti-diabetic activity of HR extracts was analysed by employing non-enzymatic glycosylation of hemoglobin assay. The results of non-enzymatic glycosylation of hemoglobin assay showed an increase in the non-enzymatic glycosylation of hemoglobin in NTR and HR extracts (50–250 μg/mL). The inhibition of glycosylation was concentration-dependent increases in noted with NTR and HR extracts, and α-tocopherol, which was used as a standard. Figure 5b shows that at the highest concentration of NTR and HR extracts tested (250 μg/mL), a maximum inhibition of glycosylation was observed (70.25% and 82.75%, respectively). Effective treatment for diabetes can result from using the bioactive elements present in plant extracts [45]. Polyphenolic compounds have potent inhibitory effects on α-amylase and α-glucosidase [46]. 

Inflammation is a biological response to harmful stimuli such as pathogens that cause tissue and cellular damage. Phenolic compounds that can interfere with these mechanisms by preventing a prolonged inflammation could be useful for human health. Lipoxygenase inhibitors are involved in numerous inflammatory diseases, such as cancer, asthma, leukemia, lymphoma, autoimmune disorders and they can increase the immune response to viral and bacterial infections [47]. The anti-inflammatory capacity was evaluated by lipoxygenase activity (Figure 5c). HR extracts showed a maximum activity which is about 73.75% higher than the activity of NTR extracts (61.55% inhibition). Comparable results were obtained in peanut culture where HRs can produce useful compounds with anti-inflammatory activities [48]. Similarly, essential oils of *Leonurus sibiricus* HRs exhibited activity in the in vitro 5-lipoxygenase assay in the inflammation process [49]. The promising anti-lipoxygenase activity of HR extracts may be associated to the presence of flavonoids. HR extracts can inhibit the membrane stabilization 84.75%, and it is near to the standard aspirin (89.52%, Figure 5d). Denaturation of proteins leads to inflammation. HR extracts strongly inhibited the denaturation of protein in a membrane stabilization test. HR extracts showed anti-inflammatory activity in *Lopezia racemosa* due to the higher amount of campesterol derivatives [50].

**Figure 6:**
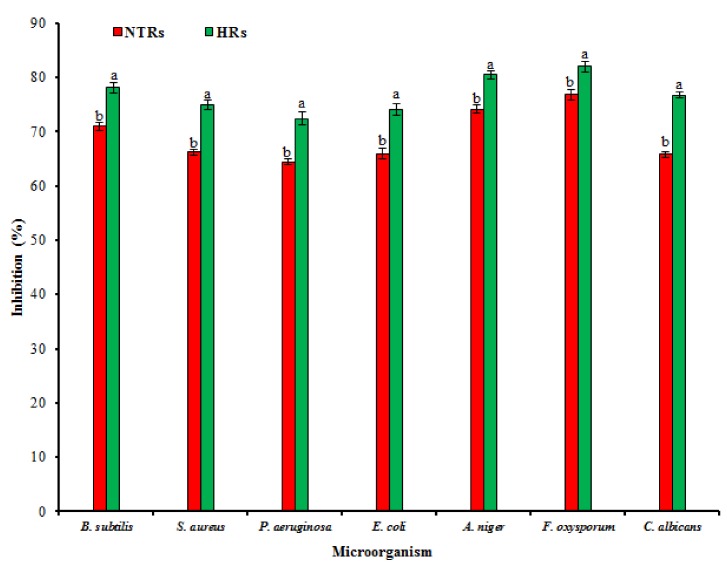
Antimicrobial activity in hairy root cultures of *Ligularia fischeri* using disc diffusion method. Statistical analysis was performed for each microbial species separately. Data represent means ± SD of three replicates. If followed by different letters, values are significantly different at *p* ≤ 0.05.

HR and NTR extracts exhibited variable antimicrobial activity measured by zones of growth inhibition (Figure 6). Maximum activity was observed with both Gram-positive and Gram-negative bacteria in HRs than NTRs. HR extracts showed more distinct activities against Gram-positive than Gram-negative bacteria, which is in accordance with studies on the antibacterial activity of *Catharanthus roseus* [51]. These results are expected due to the absence of a lipopolysaccharide membrane surrounding the cell wall of Gram-positive bacteria allowing increased permeability of *Hypericum* antimicrobial metabolites into cells [52]. Numerous studies have confirmed that HRs showed more significant antibacterial and antifungal activity than NTR extracts [10,52]. Our results confirm that the HR extracts have shown potent antimicrobial activity against clinically significant microorganisms.

**Figure 7:**
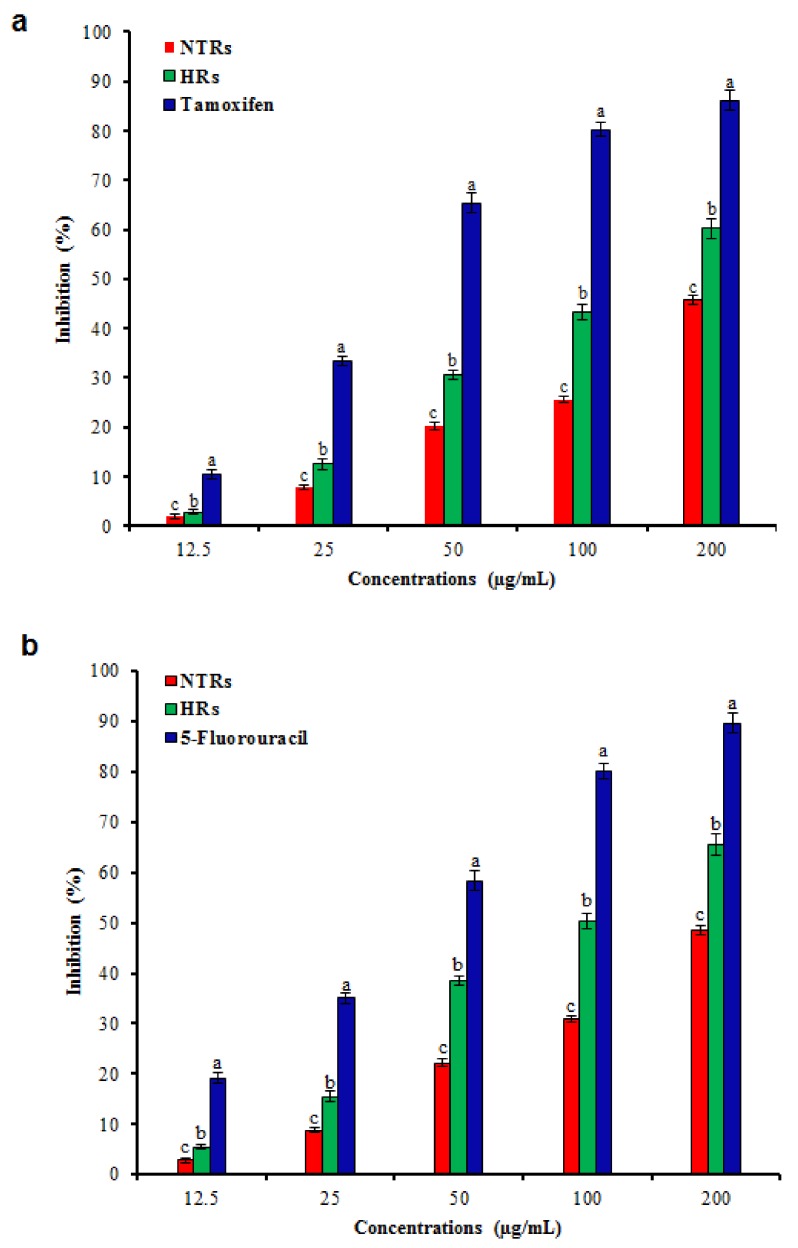
Cell viability of MCF-7 and HT-29 cell lines in hairy root cultures of *Ligularia fischeri*. (**a**) MCF-7, (**b**) HT-29. Data represent as means ± SD of three replicates followed by different letters, are significantly different at *p* ≤ 0.05.

Screening for cytotoxic activity of HR and NTR extracts against MCF-7, and HT-29 cancer cells was investigated. The cancer cells were subjected to several concentrations of the HR and NTR extracts. The results showed that the percentage of cancer inhibition depends on the concentration of the extract used (Figure 7). A greater inhibition was noted at the highest extract concentration (200 μg/mL, Figure 7a,b), at which the HRs extracts exhibited high cancer inhibition whereas the NTR extracts showed less inhibition. This high cytotoxic activity in HRs may be due to the higher amount of polyphenolic compounds. Our results agreed with various earlier studies which demonstrated that the HRs displayed higher cytotoxic activities compared to the NTR extracts [39,50,53]. 

## 3. Materials and Methods

### 3.1. Induction and Proliferation of Hairy Root Cultures (HRCs)

Seeds of *L. fischeri* were obtained from Asia Seed Co., Ltd. (Seoul, Korea) and seeded in trays with 200-cell plugs with the commercial medium and maintained in a controlled-glass house at 25 °C/18 °C day/night. After 3 weeks, leaves, petioles, and roots were excised and treated with sodium hypochlorite at a concentration of 1% (*v/v*) for 10 min and washed four times with sterilised water. Later leaves, petioles, and roots were infected with *Agrobacterium rhizogenes* (KCTC 2703) for 30 min. *A. rhizogenes* was allowed to induce gene transfer into the explant cells for 3 day on semisolid MS [54] medium and cultured in the dark conditions at 25  ±  1  °C. After incubation, explants were washed with cefotaxime (300 mg/L) to eradicate *A. rhizogenes*. HRs production was observed from the different part of explants after two weeks of inoculation. The HRs (500 mg FM) were separated from the explants and inoculated to an MS liquid medium supplemented with cefotaxime (300 mg/L). After a 3-week interval, HRs were sub-cultured in MS liquid medium with gradually reducing the concentrations 200, and 100 mg/L cefotaxime. Finally, the transformed HRC was maintained in 250-mL flasks with 50 mL of cefotaxime free MS liquid medium. Flasks were kept in a rotary shaker (Hanyang Science Equipment, Seoul, Korea) (100 rpm at 25 ± 1 °C under a 16 h light/day). The HRs were sub-cultured every 2 weeks into fresh medium. Non-transgenic roots (NTRs) were used from in vitro plants that were developed in the hormone-free MS liquid medium. The genetic transformation of HRs with T-DNA of *A. rhizogenes* confirmed using a polymerase chain reaction (PCR). For this study genomic DNA was obtained from putative transgenic (HR) lines and NTRs by using a DNeasy Plant Mini Kit (Qiagen, Seoul, Korea) rendering to manufacturer’s instructions. PCR amplification was done using a PCR (PerkinElmer, Waltham, Massachusetts, USA). PCR conditions and the *rolC* gene was using specific primers according to our previous report [10]. 

### 3.2. Growth Kinetics of HRCs

Growth was assessed in four different culture media; MS [54], B5 [55], LS [56], NN [57] and, along with sucrose at different concentrations (10, 20, 30, and 40 g/L), and various interval of time (7, 14, 21, 28, and 35 day) was examined to find out the biomass accumulation. HRC were maintained on a shaker at 100 rpm with the same culture conditions. All cultures were harvested after a growth of 28 day, HRs were washed in disinfected water, blotted on sterile tissue paper and calculated the fresh mass (FM). The dry mass (DM) of HRs was noted after drying in a hot air oven at 40 °C (48 h) till a constant weight was attained.

### 3.3. Quantitative Analysis of Phenolic Compounds 

#### 3.3.1. Total Phenolic Compounds (TPC)

TPC of the HR and NTR extracts was estimated using the Folin-Ciocalteu (FC) method [58,59]. The sample extracts (100 μL, 100 mg/mL) was mixed with deionized water (3.0 mL) and FC reagent (200 μL). After 6 minutes, sodium carbonate solution (600 μL, 20%) was allowed to stand for 2 h at room temperature. The optical density (OD) was read at 765 nm using the UV–visible spectrophotometer (Mecasys, Daejeon, Korea). TPC was calculated as mg of gallic acid equivalent (GAE)/g by using an equation from the gallic acid calibration curve. 

#### 3.3.2. Total Flavonoid Contents (TFC)

TFC of the HR and NTR extracts was assessed using a previously described procedures [58,59]. The sample extracts (100 μL, 100 mg/mL) was mixed with the reaction mixture (10% aluminium chloride 100 μL, 1 M potassium acetate 100 μL and 4.3 mL distilled water) and mixed well. The mixture was kept at 30 °C for 30 min. The OD was measured at 415 nm in a spectrophotometer (UV–Vis) and displayed the values as mg of quercetin/g DM.

#### 3.3.3. Extraction of Phenolic Compounds

Lyophilized and powdered samples of HRs and NTRs were extracted using an earlier described method [59]. The sample (1 g) was mixed to acetonitrile (10 mL), and 2 mL hydrochloric acid (2 N) and the mixture was stirred in a rotary shaker for 2 h at room temperature. The mixture samples was filtered using Whatman filter paper (No. 42) and the filtrates were dissolved in 10 mL of MeOH (80%) and filtered using a 0.45 μm membrane. This filtrate was used for UHPLC analysis.

#### 3.3.4. UHPLC Analysis of Phenolic Components 

These analyses were performed on UHPLC system (Thermo Accela, New York, NY, USA). The separation of compounds was accomplished using a HALO C18 column (2.7 μm, 2.1 mm × 100 mm) and the absorbance was measured at 280 nm. The mobile phases were 0.1% glacial acetic acid (GAA) in distilled water (Solvent A) and 0.1% GAA in acetonitrile (Solvent B). Each sample (4 μL) was injected, with each run using the following linear gradient solvent (0 min, 92% A; 0–2.2 min, 90% A; 2.2–5 min, 85% A; 5–7.5 min, 84.5% A; 7.5–8.5 min, 82.2% A; 8.5–13 min, 55% A; 13–14 min, 100% B; and 14–15 min, 92% A). The flow rate was maintained at 500 μL/min. Solutions of pure myricetin, quercetin, kaempferol, rutin, naringenin, biochanin A, formononetin, caffeic acid, *p*-coumaric acid, ferulic acid, *m*-coumaric acid, *o*-coumaric acid, chlorogenic acid, gallic acid, protocatechuic acid, *β*-Resorcylic acid, syringic acid, vanillic acid, gentisic acid, salicylic acid, homogentisic acid, veratric acid, resveratrol, pyrogallol were used as standards (25, 50, 100 and 150 mg/mL). The standards were obtained from Sigma–Aldrich (St. Louis, MO, USA) and dissolved in MeOH and analyzed before sample analysis. Each of the quantified phenolic compounds was expressed as μg/g [59]. 

### 3.4. Determination of Pharmacological Activities 

#### 3.4.1. Preparation of Extracts 

Lyophilized and powdered samples (1 g DM) of HRs and NTRs was extracted with 30 mL of MeOH (95%) and maintained at 23 °C for 24 h with constant shaking used rotary shaker at 110 rpm. Consequently, the solution was passed through Whatman filter paper (No. 42) and evaporated to dry the filtrate using a rotary evaporator. The extract was then dissolved with methanol and kept at 4 °C for following analyses on pharmaceutical activities.

#### 3.4.2. Antioxidant Activities

##### Radical Scavenging Capacity

Free radical scavenging potential was measured according to the methods [10,59]. The sample extracts (100 μL) was mixed with a solution of 1 mL 2,2-diphenyl-1-picrylhydrazyl (DPPH) solution and kept in the dark at room temperature for 30 min, and OD was read at 517 nm using a UV–visible spectrophotometer:

Inhibition (%) = (OD*_blank_* − OD*_sample_*/OD*_blank_*) × 100 (1)

##### Nitric Oxide Scavenging Capacity 

Nitric oxide radical scavenging assay was determined using the method [43]. The HRs and NTRs extracts were prepared from a 10 mg/mL ethanol. The sample extracts (100 μL) was mixed with the 10 mM sodium nitroprusside and kept at 25 °C for 2 h. The sample extracts was mixed with freshly prepared Griess reagent. The OD was observed at 546 nm:

Inhibition (%) = (OD*_blank_* − OD*_sample_*/OD*_blank_*) × 100 (2)

##### Reductive Potential

Reduction power of ferric was determined using reported methods [10,59]. The sample extracts (100 μL) was mixed with 2.5 mL sodium phosphate buffer (200 mM), and 2.5 mL potassium ferricyanide (1%) was added to these solutions and incubated at 50 °C for 20 min. Then, 2.5 mL trichloroacetic acid (10%) was added, and the mixture was centrifuged for 10 min at 650 g. After centrifugation, the supernatant (2.5 mL) with 2.5 mL distilled water, and 0.5 mL ferric chloride (0.1%) was vortexed well, and the OD at 700 nm. 

##### Phosphomolybdenum Method

Antioxidant potential was assessed using the phosphomolybdenum method, as formerly reported [10,59]. The sample extracts (100 µL, 1 mg/mL) was mixed with 1 mL of phosphomolybdenum reagent (4 mM ammonium molybdate, 0.6 M sulfuric acid and 28 mM sodium phosphate) and then incubated at 95 °C for 90 min in a thermal block and then cooled to 23 °C for a few mins and the OD was read at 695 nm. 

##### Chelating Effects on Ferrous Ions

The chelating effects ferrous ion was evaluated as a reported method [19]. Briefly, 1 mL of the sample extracts (250 µg/mL) was mixed with a solution of 2 mM FeCl_2_ (0.05 mL). The reaction mixture was started by the adding of 5 mM ferrozine (0.2 mL). Then, the mixture was shaken forcefully and maintained at 23 °C for 10 min and the OD at 562 nm:

Inhibition (%) = [(OD*_blank_* − OD*_sample_*)/OD*_blank_*] × 100 (3)

#### 3.4.3. Antidiabetic Activity

##### Inhibition of α-amylase Activity

The inhibition potential was attained using the dinitrosalicylic acid (DNSA) method slight modifications [43,60]. The trial mixture contained 500 μL of 0.02 M Na_3_PO_4_ buffer having *α*-amylase solution (1 U/mL) sample extracts at 20–100 μg/mL and mixed with 100 mL of starch (1%) and incubated for 20 min at 37 °C. The reaction was over by adding 500 μL of DNSA reagent and then incubated in a boiling water bath for 10 min, and the OD was measured at 540 nm.

Inhibition (%) = [(OD*_blank_* − OD*_sample_*)/OD*_blank_*] × 100 (4)

##### Non-Enzymatic Glycosylation of Hemoglobin Activity

Non-enzymatic glycosylation of hemoglobin activity was described earlier with some modifications [60]. Briefly, 1 mL of hemoglobin (0.06%), 5 µL of gentamycin (0.02%), 1 mL sample extracts (1 mg/mL) and 1 mL of glucose solution 0.2% was mixed. The mixture was kept at 37 °C in dark conditions for 3 days and OD at 443 nm. α-Tocopherol was used as a standard drug having similar concentration as that of extract sample solutions. 

Inhibition (%) = (OD*_blank_* − OD*_sample_*)/OD*_blank_*] × 100 (5)

#### 3.4.4. Anti-inflammatory Activity

##### Lipoxygenase Assay

The lipoxygenase activity was determined according to the previous method [47]. Briefly, 200 µL mixture contained 160 µL sodium phosphate buffer (100 mM, pH 8.0), 10 µL sample extracts (25 to 100 µg in 100 mM Tris buffer pH 7.4) and 20 µL 5-lipoxygenase enzyme. The contents were preincubated for 10 min at 25 °C. The reaction was started by the adding of 10 µL linoleic acid solution as a substrate. After 6 minutes, the absorbance was noted at 234 nm. All reactions were achieved in triplicates in 96-well microplate reader (BioTek, Seoul, South Korea). The positive and negative controls were included in the experiment. 

Inhibition (%) = [(A*_control_* − A*_sample_*)/A_c*ontrol*_] × 100 (6)

##### Albumin Denaturation Inhibition Assay

Inhibition of albumin denaturation was determined according to the method [43]. The reaction mixture is consisting of the sample extracts at different concentrations and 1% BSA (aqueous solution). The reaction mixture was then kept in an incubator for about 20 min at the temperature of 37 °C, and then the incubated solution was heated at 51 °C for 20 min. The turbidity of the mixture was then estimated at 660 nm by cooling the mixture:

Inhibition (%) = [(A*_control_* − A*_sample_*)/A*_control_*] × 100 (7)

#### 3.4.5. Antimicrobial Activity

HR and NTR extracts were examined for their antimicrobial activity using a reported method [18]. The microorganisms of *Staphylococcus aureus, Bacillus subtilis* (Gram-positive) and *Pseudomonas aeruginosa*, *Escherichia coli* (Gram-negative bacteria) and fungus (*Aspergillus niger*, *Fusarium oxysporum* and *Candida albicans*) using disc diffusion method [18,59]. Concisely, 100 mL of culture bacterial cells (10^8^ CFU/mL) and fungi (10^4^ spores/mL) were spread onto a medium of nutrient agar and potato dextrose agar, respectively. Microbial inhibition potential (%) was calculated based on previous methods [18,59]. 

#### 3.4.6. Anticancer Activity

Cell viability was assessed using an MTT colorimetric method [59]. The cancer cell lines (HT-29 and the MCF-7) were used for the cytotoxicity selection of the HR and NTR extracts. Cell lines were added to 96-well plates (5 × 10^3^ cells well^−1^) and treated for 48 h within the samples (12.5, 25, 50, 100, and 200 μg/mL). The cells (control) were treated with DMSO (1%), and cells were treated with the MTT reagent (20 μL well). For all experiments, the cells were incubated at 37 °C for 4 h, and then the DMSO (200 μL) was added to all wells to dissolve the formazan crystals. The observation was read using a microplate reader at 492 nm absorbance.

Inhibition (%) = (A*_sample_*/A*_control_* × 100) (8)

### 3.5. Data Analysis

All the trials were done in triplicate with mean ± standard deviation (SD). Each experiment was repeated twice and the level of significance at *p* ≤ 0.05. Means were separated using Duncan’s multiple range test.

## 4. Conclusions

This is the first study concerning the production of polyphenolic compounds with antioxidant, antidiabetic, antimicrobial, anti-inflammatory and anticancer activities by *L. fischeri* transgenic lines induced by *A. rhizogenes*-mediated transformation. The HRs were rapidly grown in MS liquid medium supplemented with 30 g/L sucrose. The HRs produces a significantly higher amount of biomass (9.0-fold) than NTRs. HRs showed great biosynthetic potential for the accumulation of total phenolic and flavonoid contents. HRs accumulated significant quantities of flavonols and hydroxybenzoic and hydroxycinnamic acids than NTRs. In addition, HR extracts exhibited improved pharmaceutical potential measured as antioxidant, antidiabetic, antimicrobial, anti-inflammatory and anticancer activities. HRC are a great alternative to improve the production of polyphenolic compounds in *L. fischeri*. It can be concluded that the *rolC* gene can be potentially used to enhance phenolic compounds and pharmaceutical properties. Therefore, we believe that the present protocol could be useful for the industrial production of polyphenolic compounds and their uses for pharmaceutical activities concerned with significant health benefits using *L. fischeri* hairy root cultures. 

## Figures and Tables

**Table 1:** Major phenolic compounds were identified by UHPLC analysis in the hairy roots of *Ligularia fischeri*.

No.	Phenolic Compounds (μg/g DM)	Control Roots (NTRs)	Hairy Roots (HRs)
	*Flavonols*		
1	Myricetin	1964.35 ± 2.0 ^a^^,z^	2379.50 ± 3.0 ^a^^,y^
2	Quercetin	409.20 ± 3.5 ^b^^,z^	511.25 ± 4.0 ^b^^,y^
3	Kaempferol	87.25 ± 2.2 ^g^^,z^	115.50 ± 2.0 ^j^^,y^
4	Rutin	97.00 ± 1.0 ^f^^,z^	135.25 ± 2.0 ^f^^,y^
5	Naringenin	21.95 ± 1.0 ^m^^,z^	25.00 ± 1.0 ^p^^,y^
6	Biochanin A	4.30 ± 1.0 ^p^^,z^	10.00 ± 1.0 ^s^^,y^
7	Formononetin	12.30 ± 1.2 ^o^^,z^	21.85 ± 1.0 ^q^^,y^
	**Total**	**2596.35 ^z^**	**3198.35 ^y^**
	*Hydroxycinnamic acids*		
8	Caffeic acid	139.35 ± 2.0 ^d^^,z^	175.50 ± 2.0 ^d^^,y^
9	*p*-Coumaric acid	80.15 ± 1.5 ^h^^,z^	105.10 ± 1.0 ^k^^,y^
10	Ferulic acid	81.35 ± 1.0 ^h^^,z^	121.50 ± 1.5 ^i^^,y^
11	*m*-Coumaric acid	5.45 ± 1.1 ^p^^,z^	10.10 ± 1.0 ^s^^,y^
12	*o*-Coumaric acid	17.55 ± 1.0 ^n^^,y^	16.00 ± 1.0 ^r^^,z^
13	Chlorogenic acid	72.95 ± 2.0 ^i^^,z^	130.50 ± 2.0 ^g^^,y^
	**Total**	**396.80 ^z^**	**558.70 ^y^**
	*Hydroxybenzoic acids*		
14	Gallic acid	88.35 ± 1.5 ^g^^,z^	105.50 ± 1.0 ^k^^,y^
15	Protocatechuic	66.85 ± 2.0 ^j^^,z^	95.70 ± 1.0 ^l^^,y^
16	*β*-Resorcylic	25.35 ± 1.0 ^l^^,z^	33.50 ± 1.0 ^o^^,y^
17	Vanillic acid	26.60 ± 1.2 ^l^^,z^	41.59 ± 1.0 ^n^^,y^
18	Syringic acid	51.45 ± 1.4 ^k^^,z^	75.50 ± 1.5 ^m^^,y^
19	Gentisic acid	71.50 ± 2.0 ^i^^,z^	125.00 ± 2.0 ^h^^,y^
20	Salicylic acid	222.80 ± 2.0 ^c^^,z^	300.15 ± 3.0 ^c^^,y^
	**Total**	**552.90 ^z^**	**776.94 ^y^**
	*Other phenolic compounds*		
21	Vanillin	13.15 ± 1.0 ^o^^,z^	20.50 ± 1.5 ^q^^,y^
22	Homogentisic	18.90± 2.0 ^n^^,z^	24.00 ± 2.0 ^p^^,y^
23	Resveratrol	22.05± 1.5 ^m^^,z^	41.50 ± 1.0 ^n^^,y^
24	Veratric acid	21.70 ± 1.0 ^m^^,z^	40.20 ± 1.0 ^n^^,y^
25	Pyrogallol	122.00 ± 1.0 ^e^^,z^	145.00 ± 1.5 ^e^^,y^
	**Total**	**197.80 ^z^**	**271.20 ^y^**

Mean ± SD of three replicates. Numbers within a column ^a–s^, or row ^y–z^ followed by the same letters are not significantly different at *p* ≤ 0.05.
